# Supplementary material for: Transcriptomic Insights into Mechanisms of Early Seed Maturation in the Garden Pea (Pisum sativum L.)
Source: Cells. 2020 Mar 23;9(3):779. doi: 10.3390/cells9030779 (PMC7140803; doi:10.3390/cells9030779)
Supplement: Supplementary file 1 [file cells-09-00779-s001.zip › Supplementary materials/FileS3.zip!/FileS2/Hormones - Results/Summary Sheets - PATs/PAT report.pdf]

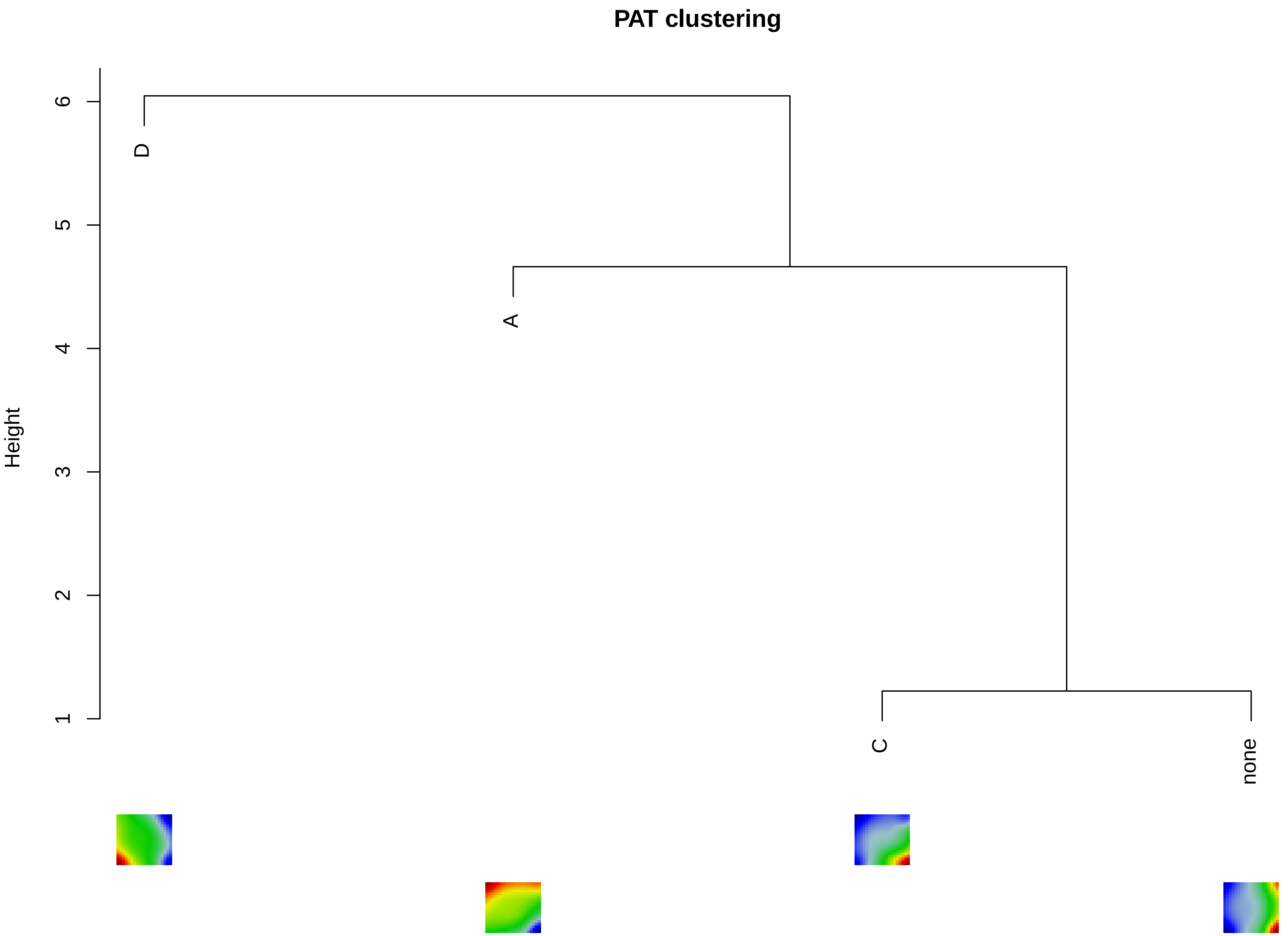
PAT clustering
Height
1
2
3
4
5
6
D
A
C
none

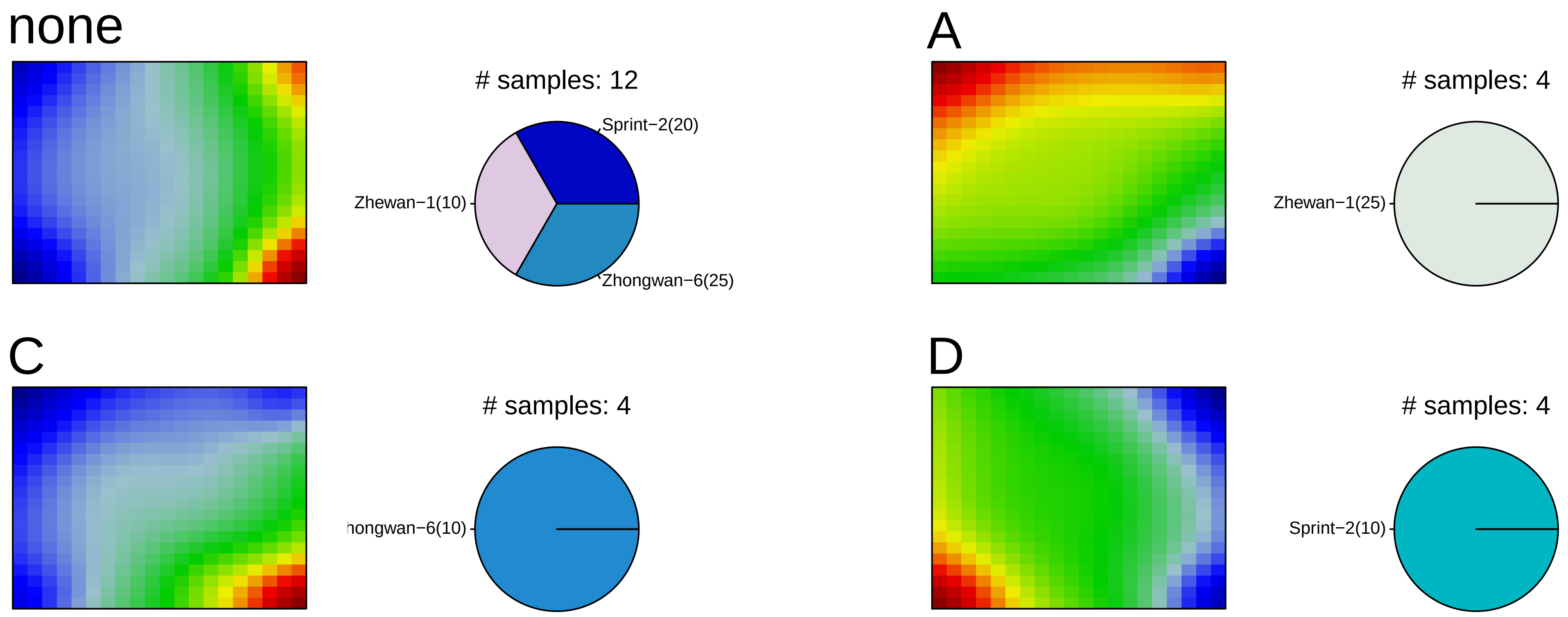
none
samples: 12
Sprint−2(20)
Zhewan−1(10)
Zhongwan−6(25)
A
samples: 4
Zhewan−1(25)
C
samples: 4
hongwan−6(10)
D
samples: 4
Sprint−2(10)

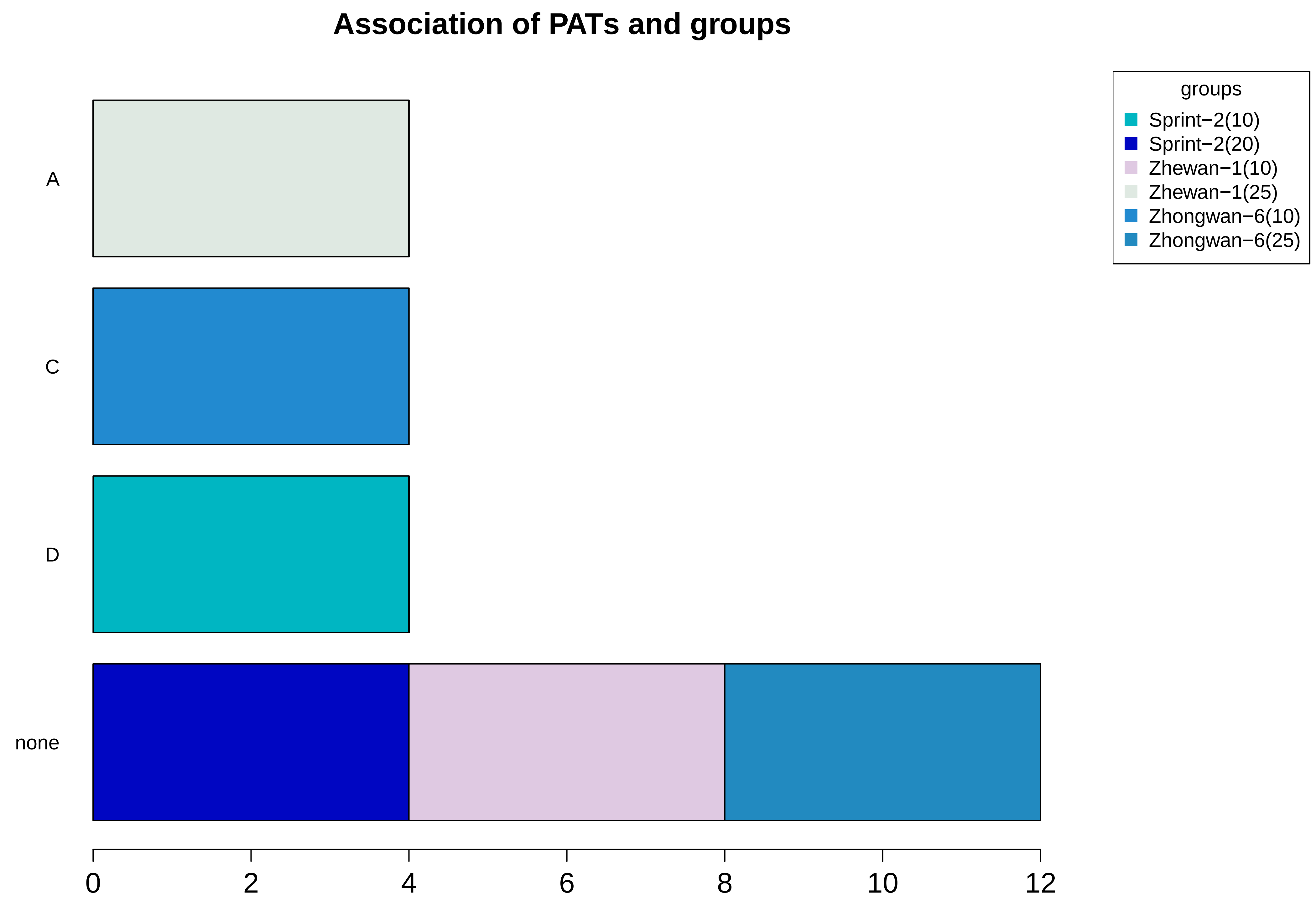
Association of PATs and groups
groups
Sprint−2(10)
Sprint−2(20)
Zhewan−1(10)
Zhewan−1(25)
Zhongwan−6(10)
Zhongwan−6(25)
A
C
D
none
0
2
4
6
8
10
12

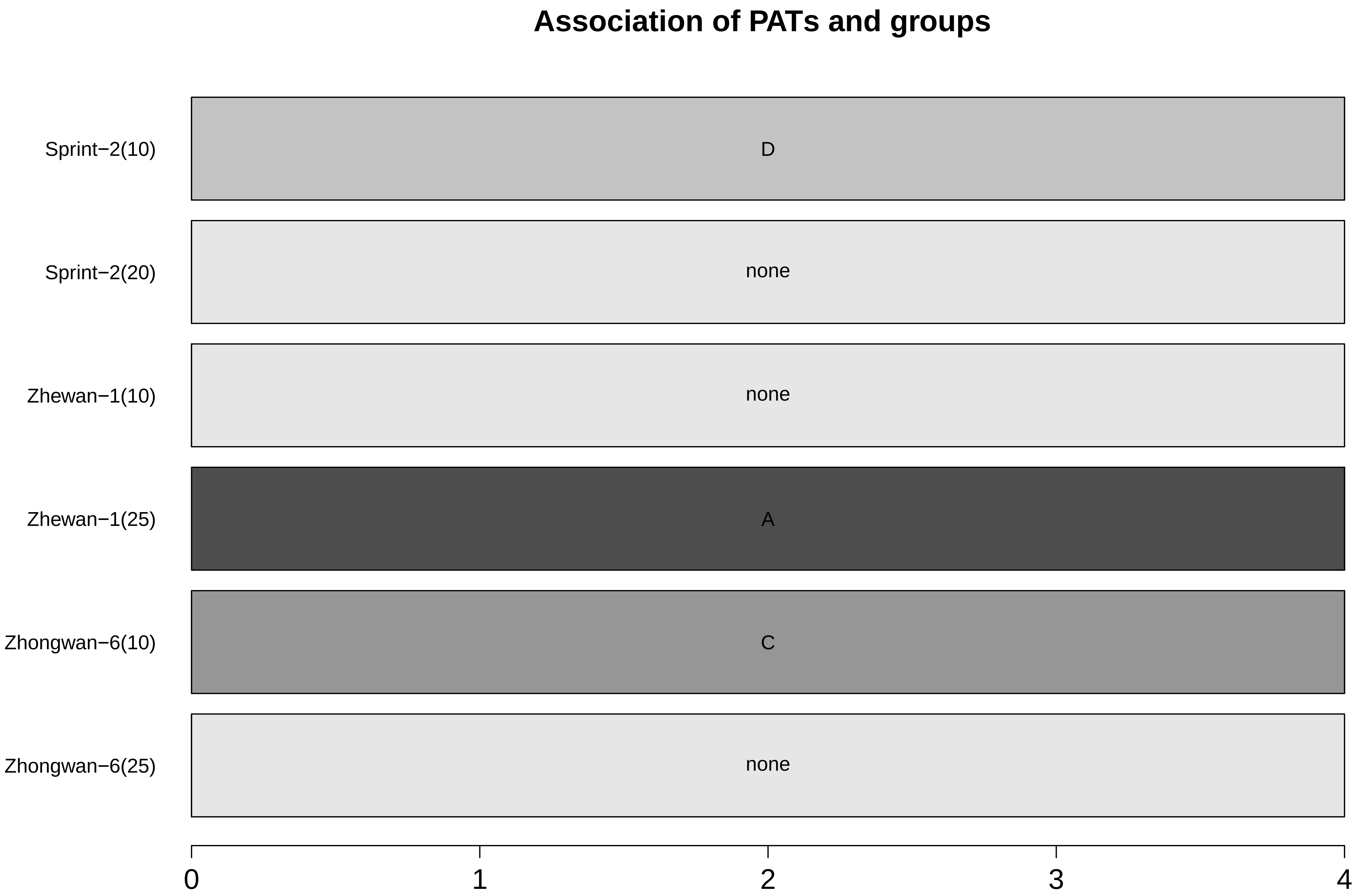
Association of PATs and groups
Sprint−2(10)
D
Sprint−2(20)
none
Zhewan−1(10)
none
Zhewan−1(25)
A
Zhongwan−6(10)
C
Zhongwan−6(25)
none
0
1
2
3
4
